# Impaired neural stem cell expansion and hypersensitivity to epileptic seizures in mice lacking the EGFR in the brain

**DOI:** 10.1111/febs.14603

**Published:** 2018-08-04

**Authors:** Jonathan P. Robson, Bettina Wagner, Elisabeth Glitzner, Frank L. Heppner, Thomas Steinkellner, Deeba Khan, Claudia Petritsch, Daniela D. Pollak, Harald H. Sitte, Maria Sibilia

**Affiliations:** ^1^ Institute of Cancer Research Department of Medicine I Comprehensive Cancer Center Medical University of Vienna Austria; ^2^ Department of Neuropathology Cluster of Excellence NeuroCure Charité ‐ Universitätsmedizin Berlin Germany; ^3^ Centre for Physiology and Pharmacology Institute of Pharmacology Medical University of Vienna Austria; ^4^ Centre for Physiology and Pharmacology Department of Neurophysiology and Neuropharmacology Medical University of Vienna Austria; ^5^ Department of Neurological Surgery UCSF Broad Institute of Regeneration Medicine University of California San Francisco CA USA

**Keywords:** Epidermal growth factor receptor, epilepsy, glutamate transporter, neural stem cells, neurodegeneration

## Abstract

Mice lacking the epidermal growth factor receptor (EGFR) develop an early postnatal degeneration of the frontal cortex and olfactory bulbs and show increased cortical astrocyte apoptosis. The poor health and early lethality of EGFR^−/−^ mice prevented the analysis of mechanisms responsible for the neurodegeneration and function of the EGFR in the adult brain. Here, we show that postnatal EGFR‐deficient neural stem cells are impaired in their self‐renewal potential and lack clonal expansion capacity *in vitro*. Mice lacking the EGFR in the brain (EGFR^Δbrain^) show low penetrance of cortical degeneration compared to EGFR^−/−^ mice despite genetic recombination of the conditional allele. Adult EGFR^Δ^ mice establish a proper blood–brain barrier and perform reactive astrogliosis in response to mechanical and infectious brain injury, but are more sensitive to Kainic acid‐induced epileptic seizures. EGFR‐deficient cortical astrocytes, but not midbrain astrocytes, have reduced expression of glutamate transporters *Glt1* and *Glast,* and show reduced glutamate uptake *in vitro,* illustrating an excitotoxic mechanism to explain the hypersensitivity to Kainic acid and region‐specific neurodegeneration observed in EGFR‐deficient brains.

**Abbreviations**
Δ: Cre‐mediated deletion
AMPAR: α‐amino‐3‐hydroxy‐5‐methyl‐4‐isoxazolepropionic acid receptor
BBB: blood–brain barrier
BCA: bicinchoninic acid assay
bFGF: basic fibroblast growth factor
ce: cerebellum
CNS: central nervous system
Co: cortex
EAAT: excitatory amino acid transporters
EGFR: Epidermal Growth Factor Receptor
EPM: elevated plus maze
FACS: fluorescent activated cell sorting
FCS: fetal calf serum
FST: forced swim test
GFAP: glial fibrillary acidic protein
Glast/Eaat1: excitatory amino acid transporter 1
Glt1/Eaat2: excitatory amino acid transporter 2
HB‐EGF: heparin‐binding EGF‐like growth factor
Hi: hindbrain
HRP: horse radish peroxidase
HS: horse serum
KA: kainic acid
LDB: light dark box test
LHRH: luteinizing hormone‐releasing hormone
Li: liver
LIF: leukemia inhibitory factor
Mb: midbrain
MK801: dizocilpine
NBQX: 2,3‐dihydroxy‐6‐nitro‐7‐sulfamoyl‐benzo[f]quinoxaline‐2,3‐dione
Nes: nestin
NMDAR: N‐methyl‐D‐aspartate receptor
Ob: olfactory bulbs
OFT: open field test
PCR: polymerase chain reaction
PDC: L‐trans‐pyrrolidine‐2,4‐dicarboxylate
PFA: paraformaldehyde
Prp^Sc^: infectious prion protein
qPCR: quantitative polymerase chain reaction
RNA: ribonucleic acid
RR: rota rod test
SPT: sucrose preference test
SVZ: subventricular zone
TGF: transforming growth factor
Tl: tail
TST: tail suspension test
VZ: ventricular zone

## Introduction

The epidermal growth factor receptor (EGFR, ErbB1) belongs to a family of receptor tyrosine kinases which are key regulators of embryonic and tumor development 1. In addition to EGFR, the family includes three additional receptors, ErbB2, ErbB3, and ErbB4. Several ligands have been described for the EGFR, the most prominent being EGF, TGFα, and HB‐EGF, while another family of ligands, the neuregulins, are known to bind to ErbB3 and ErbB4. Since there are many different ligands for the ErbB receptors, and the receptors can homo‐ and heterodimerize, a tremendous diversity of downstream signaling pathways can be initiated in response to different ligand/receptor combinations 2.

In the brain, EGFR expression increases during the late stages of embryonic development and is mainly found in proliferating and migratory regions 3, 4. Increased EGFR expression characterizes a progenitor population in the subventricular zone (SVZ) that is more prone to differentiate into the glial lineage than the neuronal lineage 5. This bias toward glial differentiation is attributed to synergistic effects of EGF and LIF (leukemia inhibitory factor) 6. Asymmetric distribution of the EGFR after mitosis of ventricular zone (VZ) and subventricular zone (SVZ) precursors seems to affect cell fate, with a predetermination of the EGFR^high^ population to an astrocytic fate 7. In the adult brain, EGFR has been shown to play a critical role in the regulation of the neurogenic niches, found in the SVZ of the forebrain and the subgranular zone of the hippocampus 8 9 10, where activation of stem cells appears to correlate with the acquisition of EGFR expression 9. Overexpression of the EGFR in neural precursor cells has been shown to result in reduced neural stem cell proliferation and self‐renewal in the SVZ 11, illustrating a critical role of EGFR signaling not only in neural stem cell maintenance but also in astrocyte differentiation.

Complete inactivation of the EGFR gene in mice produces diverse phenotypes depending on the genetic background, ranging from embryonic lethality due to placental defects through to early postnatal mortality from lung immaturity 12, 13, 14, 15. Mice surviving the first postnatal week are growth retarded and show abnormalities in skin 12, 14, 15, bone 16, 17, intestine 12 and brain 13, 15, 18 development, proving an essential role for EGFR signaling in multiple organs. Most EGFR knock‐out mice die before weaning age, although in very rare cases the mice can survive up to 1 month 12, 14. Embryonic brain development proceeds inconspicuously in EGFR knock‐out mice, but postnatally a massive degeneration of the cerebral cortex and the olfactory bulbs can be observed, which is characterized by apoptotic death of neurons as well as astrocytes 13, 18. Degenerative processes can also be detected in the thalamus but thalamic astrocytes do not seem to be affected by apoptosis and can actively respond to the neuronal degeneration by inducing reactive gliosis. Interestingly, cultured cortical astrocytes, but not midbrain astrocytes from EGFR knock‐out mice, are more susceptible to apoptosis than astrocytes from other brain regions, a phenomenon paralleled by decreased activation of Akt in cortical knock‐out astrocytes 19. Moreover, EGFR‐deficient cortical astrocytes cannot support neuronal survival in an *in vitro* coculture system between astrocytes and neurons suggesting that defects in cortical astrocytes might be responsible for the cortical neurodegeneration observed *in vivo* 19.

Astrocytes are one of the critical differentiated cell types of the brain that primarily have a protective function, forming the tripartite synapse along with the pre‐ and postsynaptic neurons. Astrocytes ensure correct homeostasis of excitatory neurotransmitters and dysregulation of astrocyte function has been associated with seizures and epileptogenesis 20. This homeostasis is largely controlled via neurotransporters, which take up excess neurotransmitters within the synaptic cleft. The primary excitatory neurotransmitter in the mammalian brain is glutamate, which is regulated by glutamate transporters (excitatory amino acid transporters; EAAT1‐5), of which Eaat2 (Glt1) and Eaat1 (Glast) are the two most prominent and expressed on astrocytes 21, 22. Glutamate transporters on glial cells have been shown to account for the majority of glutamate uptake in the brain, and impaired glutamate uptake by these transporters has been shown to contribute to the development of epilepsy 23, 24.

To investigate the role of EGFR in the adult brain, we generated two conditional EGFR‐deficient mouse models where Cre recombinase is driven by the Nestin 25 or GFAP 26 promoter. Here, we report that mice with brain‐specific ablation of EGFR do not display significant brain phenotypes but are severely susceptible to Kainic acid‐induced epilepsy. Furthermore, in the absence of EGFR we identified a defective glutamate transport activity in cortical EGFR‐deficient astrocytes proposing a neurotoxicity basis for the neurodegeneration observed in EGFR‐deleted brains. We therefore identified a critical role of EGFR signaling for the survival and functionality of cortical astrocytes and neurons through glutamate transport regulation.

## Results

### EGFR‐deficient neural stem cells show a lack of symmetric stem cell division *in vitro*

**Figure 1:**
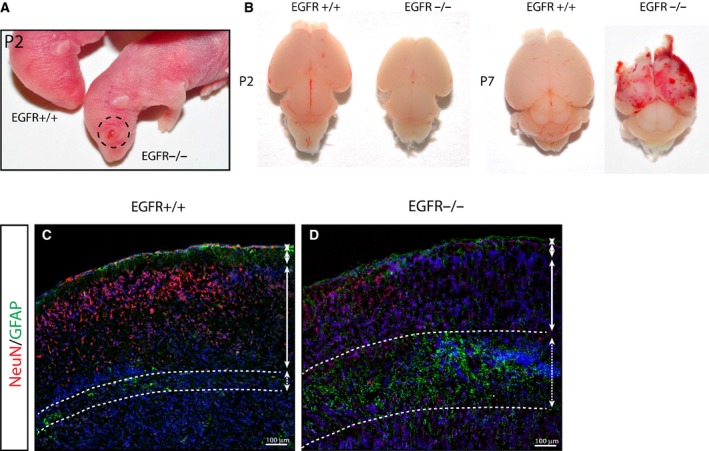
EGFR knock‐out mice display neurodegeneration (A) EGFR^−/−^ newborn mice display an open‐eye phenotype (dotted circle). (B) Whole images of brains isolated from P2 and P7 EGFR knock‐out mice (EGFR^−/−^) and littermate controls illustrate reduced brain size in EGFR^−/−^ mice. By P7 the majority (but not all) EGFR^−/−^ brains show a bloody frontal cortex. (C, D) Concomitant with a loss of NeuN+ neurons is an increase in GFAP+ cells in P6 EGFR^−/−^ mice compared with littermate controls.

**Figure 2:**
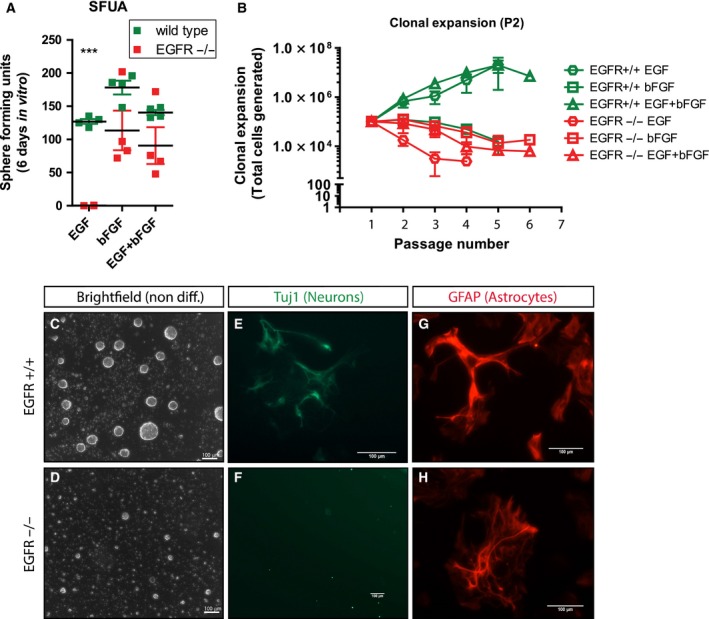
EGFR ablated neural stem cells show a lack of clonal expansion *in vitro*. (A) Primary sphere forming unit assay (SFUA). Primary EGFR‐deficient cells isolated from the cortex neurogenic niche, the subventricular zone, show reduced sphere forming potential compared to wild‐type cells when cultured with bFGF or bFGF+EGF and show complete inability to form spheres when cultured with EGF alone (*n* = 4). (B) Neurosphere clonal expansion assay. A continuation from the SFUA, prolonged cultivation of WT or EGFR^−/−^ stem‐like cells as neurospheres shows clonal expansion, and thus confirmation of symmetric stem cell division, in WT spheres (C) cultured with EGF alone (*n* = 6) or EGF+bFGF (*n* = 6) but not with bFGF alone (*n* = 5). In contrast, EGFR^−/−^ spheres (D) are unable to clonally expand in the presence of EGF (*n* = 5) or bFGF (*n* = 5). (E–H) Differentiation assay of WT and EGFR^−/−^ neurospheres. Under serum addition and growth factor removal conditions WT spheres could differentiate into neurons and astrocytes while EGFR^−/−^ spheres would only differentiate into astrocytes and not neurons. Error bars indicate SEM; *** indicates *P* < 0.0005 (*t*‐test with welches correction).

The EGFR^−/−^ mice have previously been reported to have severe developmental defects. Our EGFR^−/−^ model supports these previous data and show defects including a bulging eye phenotype (Fig. 1A) and neocortex neurodegeneration (Fig. 1B). In EGFR^−/−^ mice, neurodegeneration was frequently presented as a profound loss of NeuN+ neurons in the dorsal cortex associated with GFAP+ astrogliosis (Fig. 1C,D). EGFR signaling has been well reported to be important in the maintenance of neural stem cells 11, 27, 28, 29, 30. We wanted to investigate whether postnatal EGFR‐deficient neural stem cells were impaired, which could account for any extensive developmental defects observed throughout development. To this end we isolated cells from the cortical neurogenic niche, the subventricular zone (SVZ), from P2 EGFR^+/+^ and EGFR^−/−^ brains (*n* = 4). Initial plating of neurogenic cells under neurosphere growing conditions (termed the sphere forming unit assay, SFUA) identified a reduced neurosphere forming potential in EGFR^−/−^ mice compared with controls (Fig. 2A). Specifically, EGFR^−/−^‐derived primary cells were critically dependent on bFGF for survival and/or expansion (*P* < 0.0001), corroborating a previous study where we have shown that embryonic SVZ cells can expand in the presence of bFGF 30.

Clonal expansion of the plated cells as free‐floating neurospheres allows for the analysis of stem cell division, shown as an expanding population following sequential passaging, indicating that the primary neurogenic cells are capable of self‐renewal and proliferation. EGFR^*+/*+^‐derived neurospheres were able to clonally expand in the presence of EGF (*n* = 6) or EGF+bFGF (*n* = 6) while bFGF alone (*n* = 5) allowed only for the survival of progenitor cells (Fig. 2B,C). Interestingly, EGFR^−/−^‐derived neurospheres showed a complete inability of clonal expansion when cultured with EGF (*n* = 5) or bFGF (*n* = 5) (Fig. 2B,D). Differentiation of EGFR^*+/*+^ or EGFR^−/−^ neurospheres identified a normal capacity of WT neurospheres to differentiate into neurons and astrocytes (Fig. 2E,G) while EGFR^−/−^ neurospheres would only differentiate into GFAP+ astrocytes (Fig. 2F,H). Collectively, this data illustrate that EGFR^−/−^ SVZ‐derived cells lack the ability to undergo clonal expansion and preferentially differentiate into astrocytes *in vitro*. These results suggest that complete loss of EGFR in the SVZ neurogenic niche results in a reduced clonal expansion capacity of neural stem cells and promotes specification to a glial lineage progenitor cell.

### EGFR^ΔNes^ and EGFR^ΔGfap^ mice show growth retardation and low penetrance of cortical degeneration during early postnatal development

To better investigate the role of EGFR in adult brain development we bred mice with a conditional EGFR allele (floxed EGFR allele; EGFR^f^) 31 with two brain‐specific Cre lines with slightly different temporal and spatial expression profiles. Cre recombinase expression from the rat nestin promoter 25 was used to induce recombination of the EGFR^f^ allele in all cells of the central nervous system (EGFR^ΔNes^), beginning from approximately E14.5. Additionally, we used mice expressing the Cre recombinase under the mouse GFAP promoter 26 to trigger recombination of the EGFR^f^ allele, mainly in astrocytes (EGFR^ΔGfap^), from approximately E16.5. Cre recombinase is also active in ependymal cells and some neurons in this Cre line 26. Although the spectrum of EGFR deletion was similar with both Cre lines, the analyses that follow were performed with both lines to corroborate the findings in two different models.

**Figure 3:**
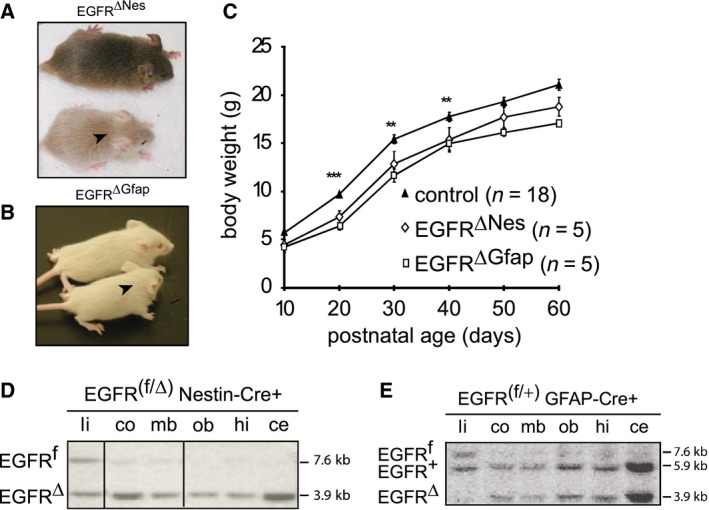
EGFR^∆^ mice are smaller than littermate controls. Gross size differences were visible between EGFR^ΔNes^ (A) and EGFR^ΔGfap^ (B) mice (arrow heads) and their respective control littermates. (C) Postnatal weight gain of wild‐type (*n* = 18) and EGFR^ΔNes^ (*n* = 5) and EGFR^ΔGfap^ (*n* = 5) mice. Results represent the mean weight ± SEM of females from at least 3 litters. Error bars indicate SEM; ***P ˂0.005, **P ˂0.05. (D, E) Southern blot analysis of genomic DNA isolated from different brain regions of an EGFR^f/∆^ Nestin‐Cre^+^ (D) and EGFR^f/+^ GFAP‐Cre^+^ (E) mouse. Because in EGFR^f/∆^ mice the ∆ allele (resulting from germline Cre deletion) was inherited from the parents, it is present in all tissues including the liver that is used as control tissue. Nes‐Cre and GFAP‐cre are not expressed in the liver and thus the floxed allele is also visible, while in the brain the floxed allele is recombined giving rise to the ∆ allele. EGFR^f/+^ mice, which have no germline deletion of EGFR, show only the floxed alleles where cre‐mediated deletion can occur.

EGFR^ΔNes^ and EGFR^ΔGfap^ mice (collectively called EGFR^Δ^) were born at the expected Mendelian frequencies and were indistinguishable from their respective control littermates at birth. However, after the first postnatal week size differences between EGFR^ΔNes^ and control mice became apparent, with EGFR^ΔNes^ mice being explicitly smaller than their littermates (Fig. 3A). Similar size differences were also observed for EGFR^ΔGfap^ mice (Fig. 3B). At weaning EGFR^ΔNes^ (*n* = 5) and EGFR^ΔGfap^ (*n* = 5) mice weighed ~ 20% less than control mice (*n* = 18) and remained significantly smaller (at 4–6 months of age Nes controls 33.43 g ± 1.97, EGFR^ΔNes^ 24.70 g ± 0.32, *P* = 0.012; Gfap Controls 32.53 g ± 1.23, EGFR^ΔGfap^ 24.94 g ± 0.85, *P* < 0.0001) (Fig. 3C). No gross neurological differences were observed between models and both strains of mice were fertile and lived a normal lifespan.

**Figure 4:**
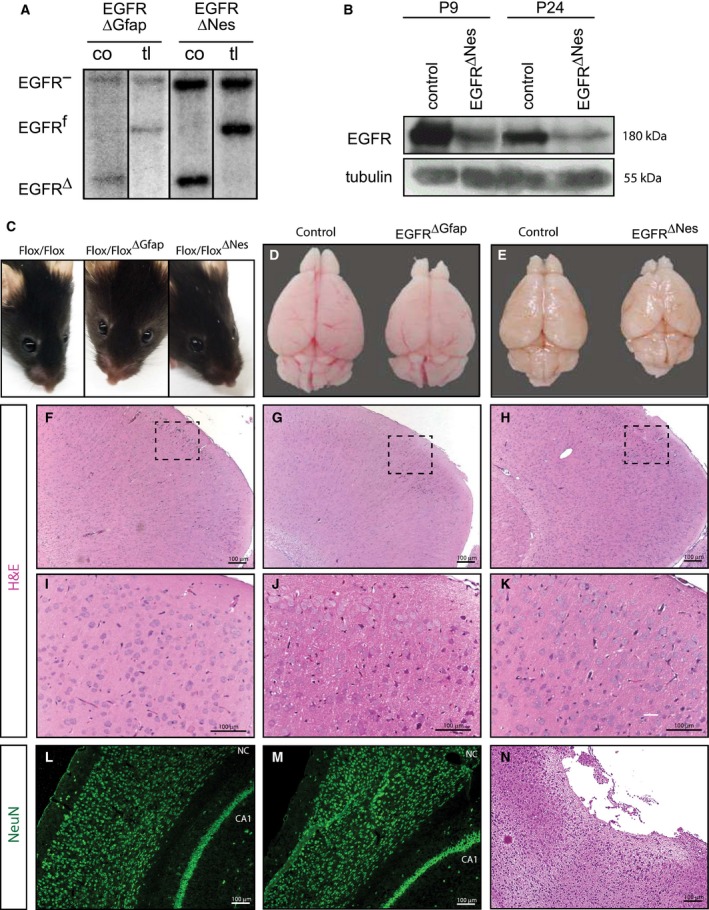
Nestin‐Cre and GFAP‐Cre‐mediated deletion of EGFR results in a grossly normal neocortex with rare neurodegeneration. (A) Southern blot analysis of EGFR recombination in cortices of EGFR^ΔGfap^ and EGFR^∆Nes^ mice at birth (P0). An EGFR‐ knock‐out allele and an EGFR flox allele can be observed as the EGFR^ΔGfap^ and EGFR^∆Nes^ mice were breed with conventional EGFR knock‐out mice to ensure Cre‐mediated deletion of the floxed allele. Tail‐DNA from the respective mice (tl) were used as controls. (B) Western blot analysis showing expression of EGFR protein in the cortices of EGFR^∆Nes^ mice at postnatal day 9 and 24. (C) No occular phenotypes were observed in adult EGFR^∆Nes^ or EGFR^ΔGfap^ mice compared to control mice. (D, E) Brains from EGFR^∆Nes^ or EGFR^ΔGfap^ are smaller than those from control littermates but show no obvious cortical degeneration. (F–K) Histological sections show normal cortical organization in EGFR^ΔGfap^ (G) and EGFR^∆Nes^ (H) cortices compared to control cortices (F); to allow a better representation of cortical lamination a higher magnification of the cortices shown in (F), (G), (H) are shown in (I), (J), (K), respectively; cortical degeneration is visible in some EGFR^∆Nes^ mice (N). (L, M) Immunofluorescent staining for NeuN in EGFR^ΔGfap^ and control cortices. Neurons (NeuN+) appear unaffected in EGFR^ΔGfap^ cortices compared with controls. NC, neocortex; CA1, hippocampal cornu ammonis area 1; I–VI, cortical layers I–VI. Abbreviations in (A) co, cortex; ce, cerebellum; hi, hindbrain; li, liver; mb, midbrain; ob, olfactory bulbs.

The midbrain, olfactory bulbs, hindbrain and cerebellum all showed recombination of the EGFR allele in both EGFR^ΔNes^ and EGFR^ΔGfap^ mice at birth (Fig. 3D,E). In addition, Southern blot analysis revealed that most of the EGFR^f^ allele is recombined in both EGFR^ΔNes^ and EGFR^ΔGfap^ cortices at birth (postnatal day 0; P0) (Fig. 4A). At P9 EGFR protein could still be detected in the cortex of EGFR^ΔNes^ mice by western blot (Fig. 4B). The EGFR^ΔNes^ model was chosen because of the more complete recombination of the conditional EGFR allele. Although the amount was clearly diminished compared to a control cortex, it was probably sufficient to guarantee basic EGFR functions. Even at weaning age (P24) residual EGFR protein was detectable in EGFR^ΔNes^ cortices (Fig. 4B). These results indicate that in the early postnatal period some EGFR protein is still present in the brain of EGFR^ΔNes^ mice, which may account for the absence of neurodegeneration.

External analysis of the eyes of EGFR^ΔNes^ and EGFR^ΔGfap^ mice identified no obvious abnormalities (Fig. 4C), which, in contrast, were always observed in EGFR^−/−^ mice (Fig. 1A). Gross analysis of brains from EGFR^ΔGfap^ (Fig. 4D) and EGFR^ΔNes^ (Fig. 4E) mice showed that these brains were smaller than those from control mice. The differences in brain size were comparable to the size differences observed in EGFR^ΔNes^ and EGFR^ΔGfap^ versus control mice but did not display any external signs of degeneration. In EGFR^−/−^ mice degeneration of the frontal cortex is macroscopically detectable in the second postnatal week, when large parts of the upper cortical layers have degenerated, usually concomitant with a bloody frontal cortex by ~ P7 (Fig. 1B) 19. To further analyze whether brain development was defective in EGFR^Δ^ mice, brains were sectioned and analyzed by immunohistochemistry. Hematoxylin and Eosin staining of sagittal sections showed that the structure of the main brain regions was comparable between EGFR^ΔNes^, EGFR^ΔGfap^ and control mice (Fig. 4F–K). In EGFR^−/−^ mice, neurodegeneration is frequently presented as a profound loss of NeuN+ neurons in the dorsal cortex associated with GFAP+ astrogliosis (Fig. 1C,D). Staining with neuronal markers did not reveal any significant differences in cortical organization and cortical lamination between EGFR^ΔGfap^ and control mice (Fig. 4L,M) indicating that no degenerative processes were occurring in the majority of these mice. Therefore, it seems that cortical development is not compromised in EGFR^Δ^ mice. However, upon analysis of more EGFR^ΔNes^ mice it became apparent that a small number of these mice (less than 5%) exhibited partial cortical degeneration with destruction of laminar patterns (Fig. 4N), similar to the degeneration observed in EGFR^−/−^ mice.

**Figure 5:**
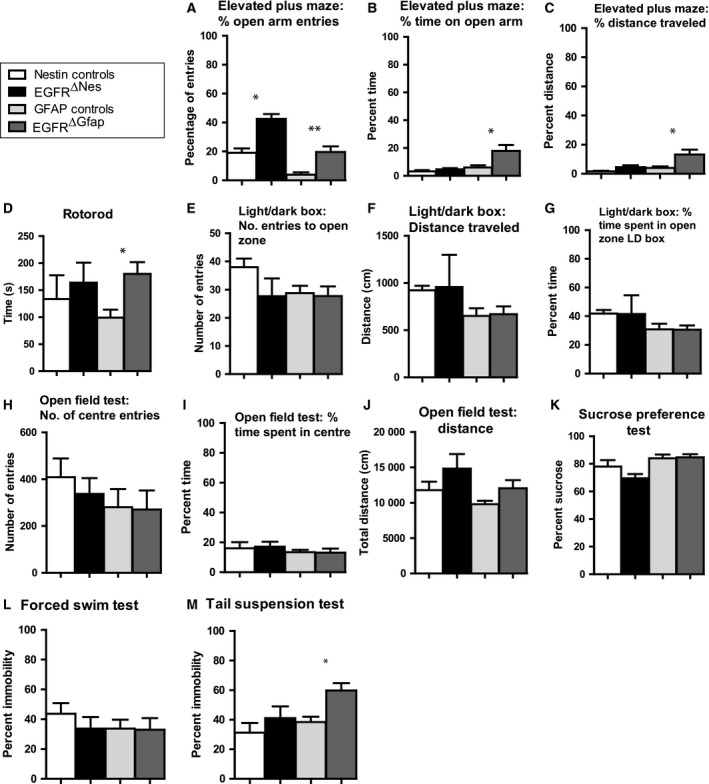
EGFR^∆^ mice display no gross behavioral differences. (A–M) Behavioral studies on EGFR^ΔNes^, EGFR^ΔGfap^ and littermate control mice. While some differences were observed between EGFR^ΔGfap^ (*n* = 13) and controls (*n* = 11) in the Elevated plus maze test (A–C), Rotarod test (D) and tail suspension test (M) the remaining studies showed no significant differences between genotypes. EGFR^ΔNes^ mice (*n* = 3) showed no statistical differences to controls (*n* = 3) in all tests save the Elevated plus maze test (A). All data are displayed as mean ± SEM; *= *P* < 0.05, **= *P* < 0.005; statistical significance calculated using one‐way ANOVA with *post hoc* analysis.

To investigate whether EGFR^Δ^ mice had any behavioral abnormalities they were subjected to an array of behavioral analyses. All tests were carried out on male adult mice (4–6 months of age). While some differences were observed between EGFR^ΔGfap^ (*n* = 13) and controls (*n* = 11) in the Elevated Plus Maze (Fig. 5A,B,C: 5.0‐fold increased percent entries in open arms, *P* = 0.004; 2.94‐fold increased percent time in open arms, *P* = 0.050; 3.28‐fold increased percent distance, *P* = 0.046), Rota Rod test (Fig. 5D: 1.8‐fold increased latency, *P* = 0.047), and Tail Suspension Test (Fig. 5M: 1.56‐fold increased percent time spent immobile, *P* = 0.014) the remaining studies showed no significant differences between groups (Fig. 5). EGFR^ΔNes^ mice (*n* = 3) showed no statistical differences to controls (*n* = 3) in all tests save the Elevated plus maze test (Fig. 5A: 2.23‐fold increased percent entries in open arms, *P* = 0.037). While single results of the behavioral analysis suggest some alterations in emotional behavior and motor coordination, these observations were not confirmed in other tests suggesting that conditional EGFR deletion in the mammalian brain does not affect behavior.

**Figure 6:**
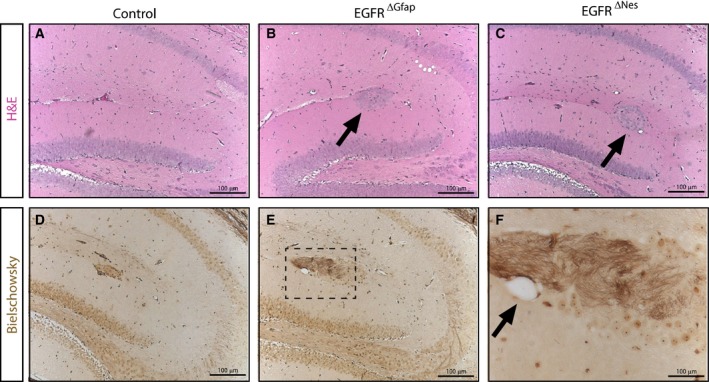
EGFR^∆^ brains show ectopic neuron formation in the hippocampus. Nests of ectopic neurons are present in the white matter of the hippocampi in EGFR^ΔGfap^ and EGFR^∆Nes^ mice. Histological sections of the hippocampi of either control (A), EGFR^ΔGfap^ (B) or EGFR^∆Nes^ mice (C). (D–F) Bielschowsky stainings of the hippocampus from an EGFR^∆Nes^ mouse. (D) and (E) represent images from different lateral positions of the same hippocampus. (F) shows a magnification of the boxed region in (E). Arrows in (B, C) indicate nests of ectopic neurons; (F) indicate neuronal plaques.

Aside from the cortex we also analyzed other brain regions affected in the EGFR^−/−^ mice. We have previously reported that the thalamus of EGFR^−/−^ mice was also affected by degenerative processes, which are accompanied by massive astrogliosis 13. Neither neuronal loss nor increased astrogliosis could be observed in the thalami of EGFR^ΔNes^ and EGFR^ΔGfap^ mice, however, closer inspection of the hippocampi revealed the presence of nests of ectopic neurons in the white matter both in EGFR^Δ^ mice (Fig. 6A–C), a phenotype previously observed also in EGFR^−/−^ mice 13. These findings were further confirmed via Bielschowsky staining which showed the presence of unmyelinated axons and neuronal plaques in the regions above the ectopic neurons (Fig. 6D‐F). Collectively these results show that EGFR^ΔNes^ and EGFR^ΔGfap^ mice have smaller brains that are generally normal on a histological scale, albeit with rare neurodegeneration.

### Absence of the EGFR in brain cells does not affect blood–brain barrier function, astrogliosis, or response to pathogenic insult

**Figure 7:**
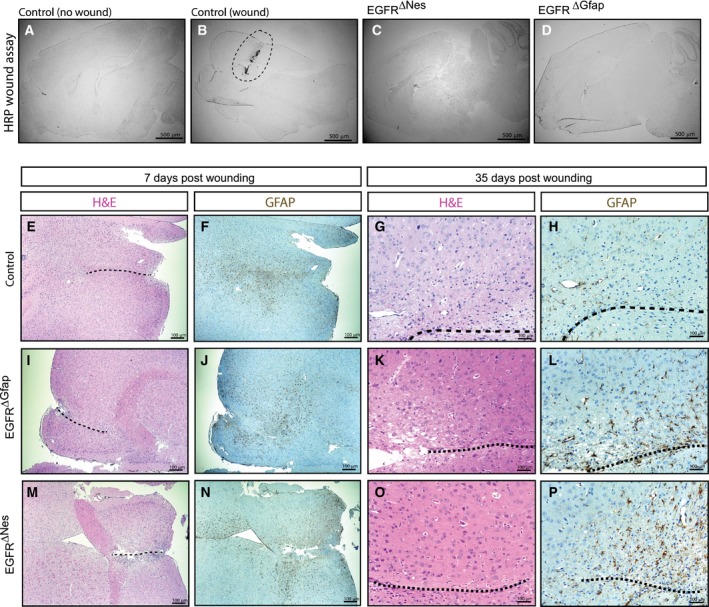
Absence of EGFR in brain cells does not impair astrogliosis or affect the response to pathogenic insults (A–D). HRP stab wound assay of EGFR^∆^ mice. No HRP‐specific substrate staining was observed in (A) control brains, (C) EGFR^ΔGfap^ brains or (D) EGFR^∆Nes^ brains but HRP‐specific substrate staining is clearly visible in (B) stab wound control brains (dotted circle). (E–P) Wound healing and astrogliosis are not impaired in EGFR^ΔGfap^ and EGFR^∆Nes^ brains; Histological sections of adult brains 7 days and 35 days postwounding (dotted lines) stained for Hematoxylin & Eosin or GFAP. Images (E–H) show a control brain, while (I–L) and (M–P) show sections from an EGFR^ΔGfap^ and an EGFR^∆Nes^ brain, respectively.

An intact blood–brain barrier (BBB) ensures brain homeostasis by efficiently preventing molecules from entering the brain 13. Astrocytes are known to be important in BBB formation by inducing endothelial cells to form the tight junctions characteristic of the BBB 32. Given EGFR plays an important role in regulating astrocyte proliferation/differentiation, we wanted to determine whether the absence of EGFR in brain cells affects BBB function. To evaluate the integrity of the BBB, EGFR^∆Nes^ mice were injected intravenously with HRP. In the case of BBB malfunction the HRP would leak into the brain and HRP substrate‐specific staining would be detectable. Mice whose BBB was opened in response to a stab‐wound injury, which were used as positive controls, showed extensive HRP substrate‐specific staining around the wound (Fig. 7B). No HRP signal was observed in the brains of EGFR^ΔNes^ (Fig. 7C), EGFR^ΔGfap^ (Fig. 7D) or control (Fig. 7A) mice, indicating that in adult mice BBB function is not affected by the absence of EGFR.

A prominent function of astrocytes is their role in response to brain injuries, a process termed reactive astrogliosis. During astrogliosis astrocytes become hypertrophic, upregulate GFAP expression and secrete different sets of molecules 33. In severe cases of trauma astrocytes proliferate and migrate to the injured region, where they initiate the formation of scar tissue. The EGFR ligands TGFα and EGF are implicated in the induction and proliferation of reactive astrocytes 34, 35. To determine whether the absence of the EGFR in the brain of adult mice results in functional impairment of astrogliosis, either by inhibiting astrocyte migration or by altering the composition of secreted factors, we analyzed the cellular response to traumatic brain injury. Adult mice older than 3 months were used to ensure the complete absence of the EGFR in the brain. Forebrain stab injuries were performed as previously described 36 and mice were sacrificed 1 and 5 weeks after injury to analyze wound healing. One week after the insult wounds were not completely closed irrespective of the genotype, and comparable numbers of infiltrating astrocytes were detected in EGFR^ΔGfap^, EGFR^ΔNes^, and control mice (Fig. 7E,I,M). Thirty‐five days after injury the wounds were closed in all genotypes and a glial scar had developed (Fig. 7G,K,O). GFAP‐positive reactive astrocytes were observable at 7 days (Fig. 7F,J,N) and still detectable around the scar at 35 days postwounding, indicating ongoing regeneration (Fig. 7H,L,P). We were not able to detect differences concerning the quality of the glial scar or the time required for healing between the genotypes. These results indicate that EGFR signaling is dispensable for astrogliosis and brain injury repair.

**Figure 8:**
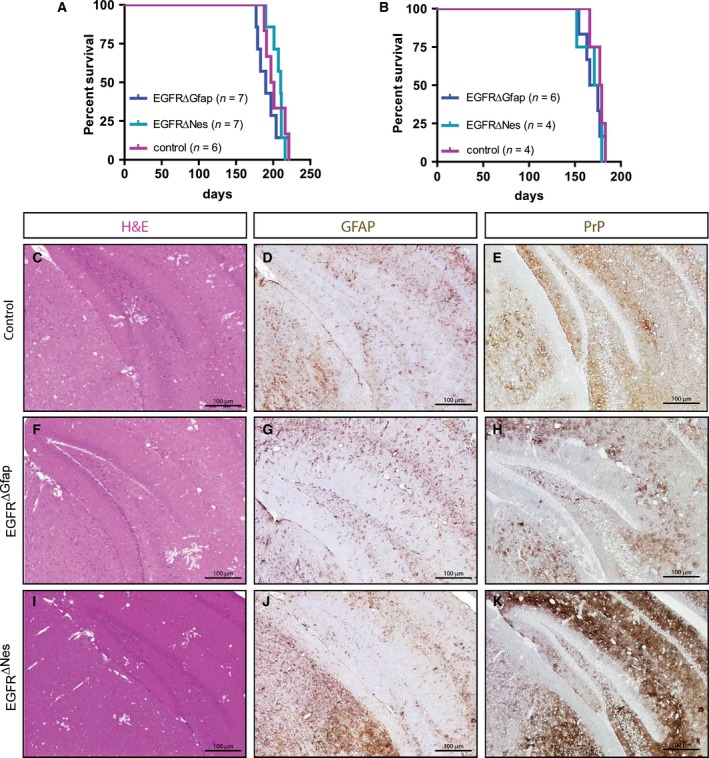
Prion infection assay. (A, B) The response to Prion‐induced insults is not impaired in EGFR^∆Nes^ and EGFR^ΔGfap^ mice. Kaplan–Meier plots showing the survival of EGFR^∆Nes^ and EGFR^ΔGfap^ mice compared to controls after intracerebral prion inoculation using either a low dose (A; *n* = 6–7) or a high dose (B; *n* = 4–6) of RML5. (C–K) Comparable neuropathological changes in the hippocampi of control (C–E), EGFR^ΔGfap^ (F–H) and EGFR^∆Nes^ (I–K) mice following pathogenic insult from prion infection. Spongiform changes were visualized with Hematoxylin and Eosin staining (C, F, I) and astrocyte responses were analyzed with GFAP staining (D, G, J). Immunohistochemistry against PrP showed equivalent staining in all genotypes (E, H, K). Scale 100 μm.

Since EGFR expression in astrocytes does not seem to be essential for acute injury responses, we next evaluated whether absence of the EGFR affects the response to a chronic pathogenic challenge in the brain. A well‐characterized model for such a challenge is prion disease, where brains of mice infected with prion protein develop pathological changes within 150–200 days after infection which, at the histological level, are manifested by accumulation of infectious prion protein (Prp^Sc^), development of astrogliosis and neuronal loss 37. EGFR^ΔGfap^, EGFR^ΔNes^, and control mice were intracerebrally infected with two different doses of the RML5 strain of murine adapted scrapie prions. Regardless of their genotype all animals showed comparable symptoms of prion disease and succumbed to the disease after the same incubation period (low dose: EGFR^ΔGfap^ 193 ± 16 days (*n* = 7), EGFR^ΔNes^ 207 ± 9 days (*n* = 7), control 202 ± 13 days (*n* = 6); high dose: EGFR^ΔGfap^ 170 ± 11 days (*n* = 6), EGFR^ΔNes^ 170 ± 13 days (*n* = 4), control 176 ± 7 days (*n* = 4)) (Fig. 8A,B). Histological analysis revealed the hallmarks of prion disease such as extensive neuronal loss (Fig. 8C,F,I), and accumulation of prion protein (Fig. 8E,H,K). Astrogliosis characterized by increased amounts of highly GFAP‐positive cells were also observed irrespective of the genotype (Fig. 8D,G,J). Together with the traumatic brain injury results these experiments indicate that EGFR function may contribute to, but is not strictly required for initiating a response to brain injuries.

### Absence of EGFR in brain cells increases susceptibility to Kainic acid‐induced epilepsy

**Figure 9:**
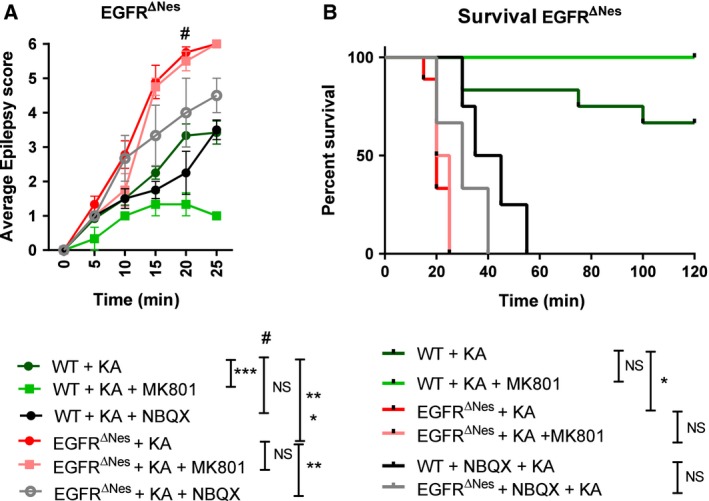
The absence of EGFR in brain cells increases susceptibility to Kainic acid‐induced epilepsy. (A) Treatment of EGFR^∆Nes^ mice with Kainic Acid (KA) resulted in significantly more severe epileptic seizures than controls, which were not ameliorated with the NMDAR antagonist MK801 or the AMPAR antagonist NBQX. (B) Treatment with KA resulted in fatalities that were not rescued with pretreatment of MK801 or NBQX. *N*‐values: WT + KA (12), WT + KA + MK801 (3), WT + KA + NBQX (4), EGFR^∆Nes^+KA (9), EGFR^∆Nes^+KA + MK801 (4), EGFR^∆Nes^+KA + NBQX (3). Error bars indicate SEM; * indicates *P* < 0.05, **P < 0.005, ***P < 0.0005. Statistical tests: A, *t*‐test; B, Log‐rank (Mantel–Cox) test.

**Figure 10:**
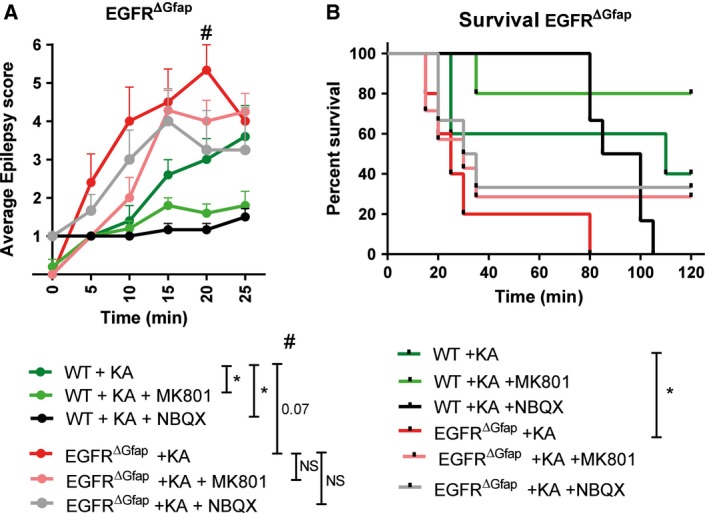
EGFR^ΔGfap^ mice display increased susceptibility to Kainic acid‐induced epilepsy and not ameliorated by glutamate receptor antagonists. (A) Treatment of EGFR^ΔGfap^ mice and control littermates with Kainic Acid (KA) resulted in severe epileptic seizures, which were not ameliorated with the NMDAR antagonist MK801 or the AMPAR antagonist NBQX. (B) Treatment of EGFR^ΔGfap^ mice with KA resulted in fatalities that were not rescued with pretreatment of MK801 or NBQX. *N*‐values: WT + KA (5), WT + KA + MK801 (5), WT + KA + NBQX (6), EGFR^∆Gfap^+KA (5), EGFR^∆NGfap^+KA+MK801 (7), EGFR^∆Gfap^+KA + NBQX (6). Error bars indicate SEM; * indicates *P* < 0.05, **P < 0.005, ***P < 0.0005. Statistical tests: A, *t*‐test; B, Log‐rank (Mantel–Cox) test.

We have previously reported that cortical neuron survival is impaired in EGFR ablated mice 19. In addition to investigating the role that loss of EGFR has with traumatic brain injury we wanted to determine if EGFR ablated brains are susceptible to neurotoxicity‐induced neuronal death. One model routinely used to investigate such phenomenon is the triggering of seizures with Kainic acid (KA), a marine‐derived amino acid that has a very high activating potential of glutamate transporters 38, 39, 40, 41. Several molecules acting downstream of the EGFR have been implicated in mediating KA‐induced neurotoxicity 42, 43. KA elicits seizures by direct stimulation of glutamate receptors, and indirectly by increasing the release of excitatory amino acids from nerve terminals 40. We wanted to determine whether the absence of EGFR signaling affects the responses to glutamatergic stimulation in the brain. Mice were injected intraperitoneally with KA at a dose of 25 mg·kg^−1^ body weight. The KA‐elicited seizures were measured over a period of 120 min and were classified according to their intensity within the first 25 min ranging from stage 1–6 as previously described 44. Stage 1 is rated as arrest of motion and the seizure level gradually increases to level 6, which is characterized by generalized tonic–clonic activity with loss of postural tone or death. KA treatment resulted in seizures in both EGFR^ΔNes^ and mice EGFR^Δgfap^, as well as their control littermates (WT). Severe seizures were observed in EGFR^ΔNes^ mice (*P* = 0.0001; Fig. 9A), and to a lesser degree in EGFR^ΔGfap^ mice (*P* = 0.07; Fig. 10A). EGFR^ΔNes^ mice seizures exhibited a progressive severity resulting in generalized tonic–clonic activity with loss of postural tone and death within 25 min after KA treatment (*n* = 9; Fig. 9A). Nine of the 12 Nestin littermate control mice survived the KA‐induced epilepsy, while 9/9 EGFR^ΔNes^ mice died within 30 min (*P* =< 0.0001; Fig. 9B). Gfap littermate control mice displayed more varied results with three of five mice surviving while five of five EGFR^ΔGfap^ mice died within 80 min (*P* = 0.035; Fig. 10B).

To determine if the seizures generated in EGFR‐ablated mice could be ameliorated by glutamate receptor antagonists we pretreated mice with either the NMDAR antagonist MK801 or the AMPAR antagonist NBQX prior to KA injection. Pretreatment with MK801 significantly ameliorated epileptic seizures in Nestin littermate control mice (median seizure score 1.5, *P* = 0.013; *n* = 3; Fig. 9A) while no significant effects were observed in EGFR^ΔNes^ mice (median seizure score 4, *P* = 0.433, *n* = 4). Pretreatment with MK801 also increased survival in Nestin and Gfap littermate control mice (0/3 deaths and 1/4 deaths, respectively) but had no effect on EGFR^ΔNes^ survival following KA‐induced epilepsy (4/4 deaths; Fig. 9B). In addition to NMDAR antagonism, pretreatment of Nestin littermate control mice with the AMPAR antagonist NBQX significantly reduced epileptic scores in control mice (15 min time point, median score 2, *P* = 0.02; *n* = 4; Fig. 9A) and significantly ameliorated KA‐induced death (4/4 deaths, *P* = 0.0013; Fig. 9B). EGFR^ΔNes^ KA‐induced seizures were similarly significantly ameliorated following NBQX pretreatment (median score 3, *P* = 0.02; *n* = 3; Fig. 9A) but survival was not significantly affected (3/3 deaths, *P* = 0.051; Fig. 9B).

Similar to Nestin littermate control mice, pretreatment of Gfap littermate control mice with NBQX (*n* = 6; *P* = 0.007) or MK801 (*n* = 5; *P* = 0.048) significantly ameliorated seizures (Fig. 10A) but had little effect on survival (Fig. 10B). Pretreatment of EGFR^ΔGfap^ mice with receptor antagonists (*n* = 7 MK801; *n* = 6 NBQX) had no significant effect on seizure severity or mouse survival following KA‐induced epilepsy (Fig. 10A,B). These results demonstrate that EGFR^ΔNes^ and EGFR^ΔGfap^ mice are much more sensitive to KA treatment than controls, with EGFR^ΔNes^ mice displaying a more aggressive epileptic response compared with EGFR^ΔGfap^ mice.

### EGFR^−/−^ cortical‐derived astrocytes show defective glutamate uptake *in vitro*

**Figure 11:**
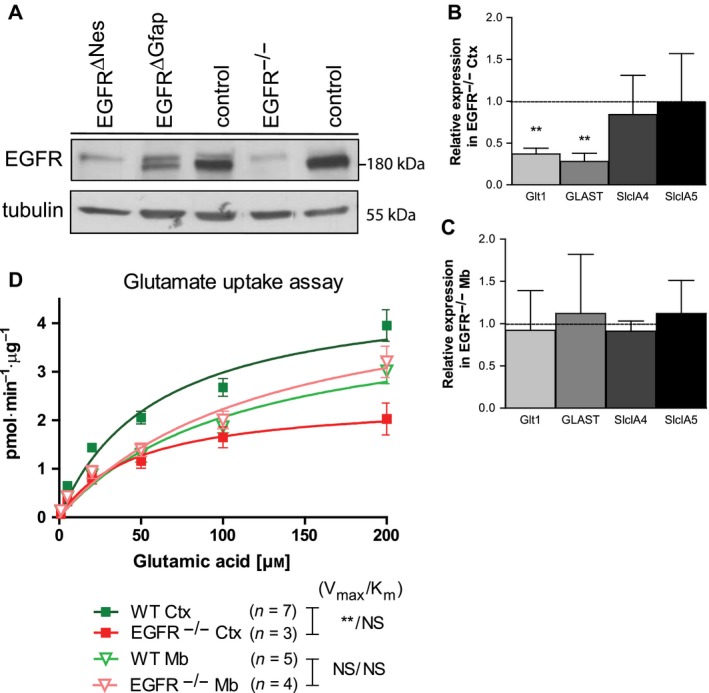
EGFR^−/−^ cortical astrocytes have deficient glutamate uptake capacity. (A) Western blot analysis of EGFR levels in cultured cortical astrocytes; EGFR protein bottom band. (B, C) Quantitative PCR analyses of EGFR^−/−^ and control astrocytes derived from the neocortex (B; *n* = 4) and midbrain (C; *n* = 4). Astrocytes were assayed for *Glt1* (*Slc1A2*), *Glast* (*Slc1A3*), *Sl1c1A4,* and *Slc1A5* and normalized to *Gapdh*. (D) Glutamate uptake assay of isolated astrocytes normalized to assayed protein levels identified a significant glutamate uptake potential in EGFR^−/−^ cortical but not midbrain‐derived astrocytes. Significant values shown in order *V*_max_ then *K*_M_. *N*‐values: WT Ctx (7), EGFR^−/−^ Ctx (3), WT Mb (5), EGFR^−/−^ Mb (4). Error bars indicate SEM; **P < 0.005; NS, not significant. Statistical tests: B/C, *t*‐test; D, unpaired *t*‐test with Welches correction.

Next, we investigated the possible mechanism responsible for the hypersensitivity to KA‐induced epileptic seizures in mice lacking the EGFR in the brain. KA leads to release of the excitatory neurotransmitter glutamate, which can directly stimulate glutamate receptors such as NMDAR1 and AMPAR1. The levels of glutamate are mainly regulated by glutamate transporters, which are abundantly expressed on astrocytes, whose primary function (among others) is to regulate extracellular glutamate levels. It has been shown that defective glutamate uptake by astrocytes can lead to neuronal death and degeneration (reviewed in 45). Thus, increased expression of glutamate receptors and/or reduced expression of transporters could account for increased KA sensitivity. Glt1 and Glast are the two most prominent glutamate transporters in the adult brain accounting for the majority of glutamate transport activity in the forebrain and cerebellum, respectively 22, 23, 46. As reported above, brains from young EGFR^Δ^ mice retain EGFR protein expression. Freshly isolated cortical astrocytes similarly identified protein expression, however, no protein was detected in EGFR^−/−^ astrocytes (Fig. 11A). We, therefore, utilized EGFR^−/−^ cortical and midbrain‐derived astrocytes in subsequent glutamate transporter analyses.

To determine if cortical and/or midbrain‐derived astrocytes from EGFR^−/−^ mice had decreased expression of glutamate transporters we investigated mRNA isolated from cultured astrocytes (*n* = 4). We identified significantly reduced expression of *Glt1* (*Slc1a2*;* P* = 0.0058) and *Glast* (*Slc1a3*;* P* = 0.001) transcripts in EGFR^−/−^ cortical‐derived astrocytes but not in EGFR^−/−^ midbrain‐derived astrocytes compared with controls (Fig. 11B,C). No differences in *Slc1A4* or *Slc1A5* transcripts were observed between groups. These data indicate that EGFR is regulating the expression of glutamate transporter transcripts in cortical astrocytes but not in the midbrain.

To investigate the hypothesis that astrocytes in EGFR ablated brains have defective/reduced glutamate uptake we utilized the well‐established glutamate uptake assay 47, 48. Primary cortical or midbrain astrocytes from P2 EGFR^−/−^ mice or littermate controls were seeded into 24‐well plates and incubated with tritium‐labeled glutamate for 10 min under controlled conditions. We found that the total uptake capacity (*V*_max_) of glutamate transporters was significantly reduced in cortical astrocytes (EGFR^−/−^ *V*_max_ 2.429 pmol·min^−1^·μg^−1^ (*n* = 3); control astrocytes *V*_max_ 4.740 pmol·min^−1^·μg^−1^ (*n* = 7); *P* = 0.001), but not midbrain astrocytes (EGFR^−/−^ *V*_max_ 4.978 pmol·min^−1^·μg^−1^ (*n* = 4); control astrocytes *V*_max_ 4.316 pmol·min^−1^·μg^−1^ (*n* = 5); *P* = 0.679) when normalized to total protein (Fig. 11D). However, the uptake function itself was not affected since the concentration of the half‐maximal transport velocity (*K*_M_) were similar for all populations (EGFR^−/−^ cortical astrocytes, *K*_M_ 45.84 μm; control cortical astrocytes, *K*_M_ 58.96 μm,* P* = 0.608; EGFR^−/−^ midbrain astrocytes, *K*_M_ 123.3 μm; control midbrain astrocytes, *K*_M_ 109.3 μm,* P* = 0.4982; Fig. 11D). These results indicate that cortical but not midbrain‐derived astrocytes have significantly lower glutamate transporter expression. Astrocytes from EGFR‐deficient mice are unable to efficiently transport the excitatory glutamate out of the synapse providing strong evidence that the observed neurodegeneration may be occurring due to excitotoxicity from excess glutamate.

## Discussion

The objective of this study was to clarify the function of EGFR in the brain by employing EGFR knock‐out (−/−) mice and mice lacking the EGFR specifically in the brain by two different Cre lines (Nestin‐Cre and GFAP‐Cre). EGFR^−/−^ mice present with an aggressive neurodegeneration shortly after birth. To determine if the neurodegeneration was associated with defective neural stem cell regulation we investigated the effect total loss of EGFR has on SVZ‐derived stem/progenitor cell division in EGFR^−/−^ mice. Utilizing *in vitro* neurosphere assays we showed that EGFR^−/−^ neurospheres did not show the cardinal stem cell traits of self‐renewal (symmetric stem cell division) or full differentiation capability illustrating that EGFR signaling is critical for these processes. These findings support previously published results that EGF and FGF growth factors are important for neural stem cell proliferation 28, 30 but report for the first time the effect complete loss of EGFR has on the neurogenic niche *in vivo* in postnatal mice. Previous studies have reported the importance of EGFR expression during neural stem cell division with differential EGFR distribution to daughter cells resulting in progenitors with different proliferative, migratory, and differentiation responses to EGFR ligands 7. Here, we show that with complete ablation of EGFR, cells derived from the SVZ show preferentially a glial‐progenitor identity, suggesting that EGFR signaling plays an important role in the switch from neural stem cell to glial progenitors.

Due to the early mortality observed in EGFR^−/−^ mice we could not utilize this model to investigate the effect EGFR loss has on neuron and astrocyte development. To this end we utlized the EGFR^ΔGfap^ and EGFR^ΔNes^ murine models. At birth, EGFR^Δ^ mice were indistinguishable from their littermates and were born at the expected Mendelian frequencies. Starting around weaning age both EGFR^Δ^ mice were consistently 20% smaller. Growth retardation was also observed in EGFR^−/−^ mice 12, 14, 15 and in mice carrying knock‐in of the human EGFR allele (EGFR^KI^) 17, whose growth retardations have been attributed to premature chondrocyte differentiation 17. However, this is unlikely to occur in EGFR^Δ^ mice where the EGFR was absent only in neural cells. Interestingly, mice lacking the EGFR family member ErbB2 in neural precursors (ErbB2^flox/flox^; Nestin‐Cre) are also growth retarded 49. Here, the growth retardation seems to be a secondary effect of a disease affecting intestinal function, which occurs as a consequence of postnatal degeneration of enteric neurons due to reduced release of growth factors by enteric glia. However, the growth retardation in ErbB2^flox/flox^; Nestin‐Cre is more severe with mice dying before 8 weeks of age 49. It is possible that enteric neurons are less affected by the absence of the EGFR. Alternatively the growth retardation observed in EGFR^Δ^ mice may be a direct consequence of the absence of EGFR signaling in the brain. Members of the EGFR family have been implicated in several functions in the hypothalamus, including locomotor activity 50. ErbB4 signaling in hypothalamic astrocytes is linked to LHRH release and delayed female sexual maturation 51. It is possible that the absence of the EGFR in hypothalamic cells may affect metabolism, leading to reduced feeding behavior or growth hormone secretion and may consequently be directly responsible for the growth retardation of EGFR^Δ^ mice. Despite their reduced size, mice lacking EGFR in the brain showed no significant changes in behavioral responses over control mice following a plethora of behavioral tests. Increased entries were recorded for EGFR^Δ^ mice in the elevated plus maze test illustrating a possible increased anxiety in these mice. This anxiety may be attributed to the loss of EGFR as it has been shown that antagonism of EGFR in cancer patients has caused increased anxiety and depression 52. However, these symptoms could also be attributable to the severe skin rash caused by EGFR inhibition 53 and remains inconclusive as to whether mice experience like‐minded states of anxiety.

Initially, we had planned to use the conditional knock‐out strategy to analyze in which neural cell‐type EGFR signaling is required to prevent the cortical degeneration. However, no EGFR^ΔGfap^ mice and only a small percentage of EGFR^ΔNes^ mice showed signs of cortical degeneration throughout their lifespan. Extensive analysis with several neuronal markers did not reveal abnormal neuronal layering of cortical layers in EGFR‐deficient brains. One phenotype observed in the brain of EGFR^−/−^ mice also found in EGFR^Δ^ brains are the ectopic neurons in the white matter of the hippocampus. The presence of these ectopic neurons indicates a function for the EGFR in neuronal migration. EGFR signaling has previously been implicated in mediating migratory processes in the brain 3, 54. The EGFR family member ErbB4 is responsible for guiding the migration of a subset of inhibitory interneurons from the subpallium to the cortex and the hippocampus 55. In the hippocampus, interneuron migration occurs along prospective white matter tracts 56 and during early postnatal development EGFR expression was observed along these migratory routes 57. Interestingly, the ectopic neurons found in EGFR^Δ^ mice are situated along this route. HB‐EGF is highly expressed in the hippocampus during late embryonic development 58. Therefore, it is possible that HB‐EGF‐induced EGFR signaling contributes to the migration of interneurons into the hippocampus. Due to the high expression levels of HB‐EGF the EGFR protein is probably degraded earlier in the hippocampus than in the cortex, and the absence of EGFR signaling during later stages of migration leads to the ectopic arrest of neurons in the white matter tract.

To analyze the function of the EGFR in the adult brain we applied several models of brain injury. These experiments were performed in adult mice where the EGFR protein could no longer be detected in the brain. In models where the major response to insults is reactive astrogliosis, like stab wounding and Prion infection, we did not observe any defects in mice lacking the EGFR in the brain. These results suggest that although EGFR signaling may play a supportive role during astrogliosis it is not strictly required for injury repair. The third model of brain injury applied was the KA seizure model. Susceptibility to Kainic Acid‐induced toxicity has been described to differ in intensity according to the genetic background 41, which might explain the different sensitivities of the Nes and Gfap control mice. Independent of the genetic background, EGFR^ΔGfap^ and EGFR^ΔNes^ mice were clearly more susceptible to KA‐induced seizures, with more severe and prolonged seizures most often leading to fatality. The excitatory mechanism occurring in an epileptic seizure has been well clarified to be caused by activation of the ionotropic glutamate receptors NMDAR, AMPAR, and the Kainate receptor 59 (reviewed extensively in 60). Complementary studies have shown that successful inhibition of ionotropic NMDAR 61, 62 and AMPAR 63 have significantly ameliorated epileptic seizures. Indeed treatment with the AMPAR and NMDAR antagonists MK801 and NBQX significantly ameliorated the KA‐induced seizures in control mice. These antagonists had, however, no effect on seizures or survival in EGFR^ΔGfap^ and EGFR^ΔNes^ mice. The reason for this might be the short half‐life of these antagonists combined with the excess glutamate present in EGFR deficient brains. The increased excitotoxicity observed in mice lacking the EGFR in the brain may be the result of diminished glutamate uptake by astrocytes, which express reduced levels of the glutamate transporters Glt1 and Glast. 19, 64, 65, 66. Glt1 and Glast have been well documented to play a critical role in regulating the amount of glutamate in the synaptic cleft between pre‐ and postsynaptic neurons 22, 23, 46, 67, 68, 69, 70. Furthermore, EGFR has been implicated in the regulation of astrocytic glutamate transporters *in vitro* and specifically, it has been shown that Glt1 expression is positively regulated by EGFR activity in cultured astrocytes 67, 68. Interestingly, we found that loss of EGFR affected the expression of *Glt1* and *Glast* exclusively in cortical‐derived astrocytes but not in midbrain‐derived astrocytes. Similarly, glutamate uptake was reduced in cortical but not midbrain derived EGFR^−/−^ astrocytes. In conjunction with our findings, studies reporting that transgenic animals overexpressing *Glt1* were less sensitive to seizure‐induced neuronal damage illustrate that amelioration of seizures is at least in part attributed to the functional activity of Glt1 68. It remains unknown as to why midbrain astrocytes are unaffected by the loss of EGFR. One explanation is the absence of a postnatal neurogenic niche, or conversely, the cortex phenotype may be associated with the presence of a neurogenic niche 27, 71, however, further studies are require to elucidate these hypotheses.

In summary, we demonstrate that complete loss of EGFR in the brain results in severe neurodegeneration and a reduction in the neurogenic potential of neural stem cells. In contrast, Cre‐mediated deletion of EGFR in the brain results in only rare neurodegeneration but severe susceptibility to KA induced epilepsy that is not ameliorated with glutamate receptor antagonists. We show that cortical but not midbrain‐derived astrocytes have reduced glutamate transporter expression and reduced glutamate transporter activity providing a mechanism of glutamate excitotoxic‐induced neuronal death. Elucidating the mechanism by which EGFR prevents glutamate‐mediated neuronal toxicity may have important implications in identifying treatment options for neurodegenerative and seizure‐associated diseases in the brain.

## Material and methods

### Animal experiments

The EGFR^f/f^ mice bred to GFAP‐Cre+ and Nestin‐Cre+ mice were maintained on a mixed genetic background (C57BL/6J × 129/Sv × CBA/J). EGFR^−/−^ mice were of pure outbred MF1 genetic background. In all experiments EGFR expressing littermates (*EGFR* ^*f/f*^ or *Cre+* or *EGFR* ^*+/*+^) served as controls to the respective EGFR deleted mice. Because of the high stability of the EGFR protein, breedings were set up to generate mice harboring always a complete knock‐out allele together with a floxed allele (*EGFR* ^*f/−*^ *)*. In this way, mice lacking EGFR protein in the brain (EGFR^ΔGfap^ and EGFR^∆Nes^ mice.) could be more efficiently generated after breeding to Nes‐Cre or GFAP‐Cre mice. Genotyping was performed by PCR as previously described 19, 72. Primer sequences can be obtained upon request. Mice were housed in a 12‐h dark/light cycle with water and food *ad libitum* and kept/bred in the animal facility of the Medical University of Vienna in accordance with institutional policies and federal guidelines. Animal experiments were approved by the Animal Experimental Ethics Committee of the Medical University of Vienna and the Austrian Federal Ministry of Science and Research. (Animal license numbers: GZ 66.009/124‐BrGT/2003; GZ 66.009/109‐BrGT/2003; BMWF‐66.009/0073‐II/10b/2010 BMWF‐66.009/0074‐II/10b/2010; BMWFW‐66.009/0200‐WF/II/3b/2014; and BMWFW‐66.009/0199‐WF/II/3b/2014).

### Behavioral tests

Animals were initially assessed by Basic Observational Neurological and physical assessment (NOB) as detailed in 73. Mice were then assayed for a number of behavioral tests including the Rota rod test (RR) 74, Open field test (OFT) 75, Light Dark Box test (LDB) 76, Elevated Plus Maze (EPM) 77, Tail suspension test (TST) 78, Forced Swim test (FST) 79, and sucrose Preference test (SPT) 80, 81. Tests were performed as detailed by references linked above. Data were recorded as percentage/number of entries (EPM, LDB, OFT), percentage/length of time spent (EPM, RR, LDB, OFT), percentage/length of distance travelled (EPM, LDB, OFT), percent sucrose (SPT), and percent immobility (FST, TST). Results analyzed with one‐way ANOVA with *post hoc* Scheffe tests.

### Southern blot

Southern blots were carried out as previously described 31 with some changes: Mouse genomic DNA was digested overnight. The samples were run on a 0.7% agarose gel and blotted overnight. Probes were labeled using the Stratagene labeling kit II (Amersham, Buckinghamshire, England) and hybridization was performed in Church Buffer at 65 °C overnight. Membranes were wrapped in Saran Wrap and films were exposed for several days.

### Western blot

Cells/tissues were homogenized with ice‐cold solubilization buffer (50 mm Hepes pH7.3, 150 mm NaCl, 10% glycerol, 1.5 mm MgCl_2_, 1% Triton X‐100, 1 mm EGTA pH8, 10 mm Na_2_P_2_O_7_, 0.001% Aprotinin, 0.001% Leupeptin, 25 mm NaF, 1 mm NaVO_3_, 1 mm PMSF, 20 mm PNPP, 10 mm sodiumpyrophosphate) and lyzed on ice for 30 min. Lysates were cleared by centrifugation for 10 min at 10 000 ***g*** and 4 °C. Lysates were shock frozen and stored at −80 °C. Protein separation was performed on SDS/PAGE Gels and proteins were transferred to nitrocellulose membranes (Amersham). Membranes were incubated with primary antibodies overnight and with secondary antibodies for an hour. Detection was performed using ECL (Amersham). Antibodies used in western blot: anti‐EGFR (Upstate 06–129, 1 : 1000), anti‐actin (Sigma, 1 : 100, St. Louis, Missouri, United States), anti‐tubulin (Sigma, 1 : 100), anti‐Rabbit HRP (DA(−/−, 1 : 10 000), anti‐Sheep HRP (DA(−/− 1 : 10 000).

### Astrocyte cultures and qPCR

Astrocytes were prepared between E18.5 and P2. Single‐cell suspensions were seeded in astrocyte medium (DMEM high glucose; 2 mm glutamine; 1 × penicillin/streptomycin; 5% fetal bovine serum (FBS); 5% Horse Serum (HS). Nearly confluent astrocyte cultures were trypsinized and reseeded in astrocyte medium. DMEM high glucose, penicillin/streptomycin, horse serum, and glutamine were obtained from Invitrogen and FBS was purchased from PAA. RNA was isolated using a QIAgen spin miniprep kit (27104) and cDNA obtained from total RNA by reverse transcription with SUPERSCRIPT First‐Strand Synthesis System (Invitrogen, Carlsbad, California, United States). qPCR analysis was done as described in 19. Assay on demand Taqman probes from Applied Biosystems (Foster City, California, United States): Glt1: Mm00441457_m1; GLAST: Mm00600697_m1; Slc1A4: Mm00444532_m1; Slc1A5: Mm00436603_m1.

### Neurosphere assays

Neurospheres were isolated as detailed in 27. Briefly, SVZ‐derived tissue was dissociated in 1 × Accutase (Life Technologies A1110501, Carlsbad, California, United States) for 7 min at 37 °C, cells centrifuged at 100 ***g*** for 5 min, supernatant removed and cells resuspended in 2 mL NS media (DMEM Thermo, 50‐124‐PA, Waltham, Massachusetts, United States; F12, Gibco 21700‐026, Waltham, Massachusetts, United States; Penicillin/Streptomycin, Gibco 15140‐122; NaHCO3, Sigma S5761; Glucose, Sigma G7021; HEPES, Sigma H4034) supplemented with 4 mL 10% BSA solution, 2 mL Penicilin/Streptomycin and 20 mL Neurocult proliferation supplement (mouse, Stem Cell Technologies 05701, Vancouver, Canada), and then filtered through a 40‐μm mesh (BD falcon 352340). Neurospheres were cultured with 20 ng·mL^−1^ EGF (Sigma E4127, St. Louis, Missouri, United States) and/or 10 ng·mL^−1^ bFGF (Preprotech 100‐18B, Rocky Hill, Connecticut, United States). For differentiation assays, neurospheres at passage 5 were seeded into 24 well plates with 10% FBS and growth factors removed. Following 6 days cells were fixed with 4% paraformaldehyde and stained via immunofluorescent methods. GFAP antibody (DAKO Z0334, Agilent Technologies, Vienna, Austria), Tuj1 antibody (Promega; G712A, Madison, Wisconsin, United States).

### Stabwound/blood–brain barrier HRP experiments

The animals were anesthetized with a combination of ketamine and xylazine according to standard protocols. Forebrain stab injuries were inflicted using a sterile No.11 scalpel blade 1 mm rostral to the bregma into the right hemisphere. The skin above the wound was closed with a suture clip. Mice were killed 7 and 35 days after the stab wounding using a lethal dose of ketamine and xylazine and were transcardially perfused with 4% paraformaldehyde (PFA). Whole mouse brains were fixed in 4% PFA, and then incubated in 20% sucrose overnight. After embedding in OCT (Sakura, Alphen aan den Rijn, The Netherlands) the tissue was shock frozen on dry ice and stored at −80 °C. For the BBB experiment HRP (5 mg/mouse) was injected with into the tail vein as detailed in 82. One hour after the HRP injection mice were killed and the brains were prepared as described for the stabwound experiment.

### Kainic acid‐induced epilepsy

Mice were intraperitoneally injected with Kainic Acid at a dose of 25 mg·kg^−1^. Mice were observed for 120 min following treatment, and kainate scores were evaluated every 5 min on a scale from 0 to 6. Seizures were scored as follows: 1, arrest of motion; 2, appearance of rigid posture; 3, myoclonic jerks of head and neck, with brief twitching movements; 4, forelimb clonus and partial rearing, unilateral; 5, forelimb clonus, rearing, falling, bilateral; 6, generalized tonic–clonic activity with loss of postural tone, often resulting in death. To study NMDAR and AMPAR antagonism we used MK801 (Tocris 0924, Bristol, United Kingdom) and NBQX (Tocris 1044), respectively. MK801 was administered 20 min prior to KA injection at a concentration of 0.2 μg·g^−1^ and NBQX was injected 10 min prior to KA injection at 30 μg·g^−1^ concentrations. All injections were done via intraperitoneal injection. Given the half‐lives of the antagonists also used in this study we underwent analysis at 20 min post‐KA injection.

### Prion infection

Mice were intracerebrally injected with the RML prion strain at doses of 10^−1^U LD50 and 10^−4^U LD50. Mice either succumbed to the disease or were euthanized when pathological symptoms became apparent. Brains were then fixed in PFA and processed for histology as described 37.

### Histology and immunohistochemistry

For routine histology brain sections were stained with hematoxylin/eosin and Bielschowsky silver stain according to standard protocols. Immunohistochemical stainings were carried out on an automated Nexus staining apparatus, following the manufacturer's guidelines. Visualization was achieved using biotin/avidin‐peroxidase (DAKO K3954) and diaminobenzidine as a chromogen. For the BBB experiment transverse brain sections were developed using DAB (diaminobenzidinetetrahydrochlorid) as HRP substrate. Sections were rinsed in PBS and stained with DAB for 20 min. Stainings were visualized using the DAB peroxidase substrate kit (Vectashield, Burlingame, California, United States), rinsed 3 × with PBS and mounted onto slides. After dehydration sections were coverslipped. For Immunofluorescent imaging, stainings were performed on 5uM cryosectioned tissue following standard protocols and if necessary a citric acid buffer antigen retrieval protocol was applied. Antibodies used: F4/80 (eBioscience 14‐4801, Thermofisher), anti‐GFAP (1 : 300, DAKO Z0334), NeuN (Millipore MAB377, Burlington, Massachusetts, United States). Fixation and staining of the brains used in the prion experiment was performed as described 37.

### Glutamate uptake assays

Glutamate uptake assays were carried out on EGFR^+/+^ or EGFR^−/−^ cortical and midbrain derived astrocytes as detailed in 72. Briefly, primary isolated astrocytes were seeded into 24‐well plates at a density of 50 000 cells/well prior to carrying out the uptake assay. Following approximately 3 days in culture (or ~ 80% confluence. Minimum 70% confluence) uptake assays were performed. Briefly, cells were washed with Krebs‐HEPES buffer (KHB: 10 mm HEPES, 130 mm NaCl, 1.3 mm KH2PO4, 1.5 mm CaCl2, 0.5 mm MgSO4, pH 7.4 adjusted with NaOH). After a 15 min preincubation step in KHB, glutamate uptake was started by adding KHB containing 0.05 μm [³H] glutamate (Perkin Elmer, Waltham, Massachusetts, United States; specific activity: 50.6 Ci/mmol) including different concentrations of unlabeled glutamate (up to 200 μm) to the cultures. Uptake was done at room temperature and stopped after 10 min by adding ice‐cold KHB. Nonspecific uptake was performed in the presence of 10 μm L‐trans‐pyrrolidine‐2,4‐dicarboxylate (PDC). Cells were washed twice with ice‐cold buffer prior to cell lysis. Radioactivity was measured using a liquid scintillation counter and 30% of the cell lysate was used for protein determination using a bicinchoninic acid assay (Bio‐Rad, Hercules, California, United States).

### Statistical analysis

Statistical analysis and graphical representation were performed using Microsoft Excel and Graph Pad Prism Software. Experiments were analyzed using t‐tests, one‐way ANOVA followed by *post hoc* Scheffe tests (for behavioral analyses), Cox–Mantel tests (for survival plots) and a *P*‐value < 0.05 was considered statistically significant. All *n*‐values shown indicate biological repeats.

## Conflict of interest

The authors declare that they have no conflict of interest.

## Author contributions

BW and JR conceived and designed the experiments, and performed most of them. JR wrote, edited, and submitted the manuscript. EG performed the FACS analyses. TS and HHS helped with the glutamate uptake assays and participated in the interpretation of the data; HHS provided the requested funding for this project part; DK and DDP performed the behavioral studies; and FH performed the stab wound and prion infection experiments. MS conceived, designed, and supervised the whole project and provided the requested funding.
